# My Experience with the Bi-Lateral Operation for Stone in the Bladder

**Published:** 1874-10

**Authors:** F. A. Stanford

**Affiliations:** Columbus, Ga.


### MY EXPERIENCE IN THE PERFORMANCE OF THE BI-LATERAL OPERATION FOR STONE IN THE BLADDER.

By F. A. STANFORD, M.D., Columbus, Ga.

*Read before the Georgia Medical Association, April, 1874.*

Having recently had the pleasure of perusing a report from Dr. Paul F. Eve, in which he gives to the profession the result of his experience in the performance of this operation, and in which its superior advantages over all other cutting operations are elaborately set forth, I consider it but proper and legitimate that I should, through this channel, submit the results of my own practice, which, though much more limited in point of numbers, is yet, I think, quite satisfactory.

Dr. Eve gives, as the result of his experience, a report of ninety operations after the bi-lateral method; of this number, he states his "mortality to be eight cases, or one in nine; this, too, without any selection of cases." Of the last forty-five of this number, his success was much greater, having lost but two cases, and these clearly not in consequence of the operation.

My own experience is limited to twenty operations for lithotomy.

From the commencement of my practice in this department of surgery, I selected the bi-lateral, as performed by that distinguished surgeon, Dupuytren, with the use of his double lithotome, as the safest for my subject, and the easiest of performance by myself. My first case was well considered, for my patient, an adult, was much worn and exhausted from a long continued suppurative cystitis; and, from being stout and robust in youth, in manhood, at twenty-three years of age, was weak and attenuated.

I removed a large stone from this patient. No shock from the operation followed, and he made a rapid and thorough recovery. My success in this case, the ease and readiness with which I entered the bladder, sought for and removed the stone, impressed me most favorably and profoundly; so much so, that I have continued this operation. Of the twenty operations that I have performed, I have lost one case. This case was that of a boy five years old, extremely exhausted at the time of discovery of the presence of the stone. Preparatory treatment accomplished no relief, and, after consultation with a number of physicians, it was decided that the operation gave the only chance for recovery. This was performed, quickly gotten through, and a large, rough stone removed. No apparent depression followed, but the patient continued to sink, and died on the third day.

I have never declined to operate in but one instance. This was in the case of a boy four years of age, in whom symptoms of stone had been present whilst a nursing child. I first saw this case in consultation, when the stone was suspected, and found to be present, upon examination. This boy was in marasmus. Preparatory treatment accomplished nothing, and he died within the week of my first visit. A post-mortem examination revealed an encysted stone, occupying the extreme fundus of his bladder. Many of my cases were regarded at the time as unfavorable for a successful operation. To these cases I have given patient, preparatory treatment before subjecting them to the knife.

I have never had but one case of alarming hemorrhage. This was promptly arrested by the introduction of a sponge tampon, perforated by gum-elastic catheter, and retained in position twenty hours.

I have never had the least appearance of urinary infiltration; generally, after the first forty-eight hours, the flow of water being divided between the wound and urethra. After this it has rapidly selected its natural channel, and been discharged at will.

Two of my patients were men with families; their wives continue to bear them children. Another, who has since the operation grown to manhood and contracted marriage, is now the father of two children.

I have operated the second time, and removed the second stone from the same bladder. I have had but one case of fistula to follow the operation; this occurred in the case above reported, and followed some months after the last operation.

In one of my cases two fistulæ had formed in the perineum long previous to any suspicion of stone. After being subjected to a variety of treatment by different physicians, I detected the presence of the stone, operated and removed it. This case was fully reported in the *New York Medical Journal*, March, 1869, with a plate showing a specimen of partially encysted stone, occupying the bas-fond of the bladder.

Chloroform, or its combination with ether, has been administered in all of these cases, no unpleasant effect following.

Cauterization of the Uterus.—Take an ordinary sponge tent and coat it with beeswax, and then roll it for some time with a knife in powdered nitrate of silver, which will sink into, and adhere to, the wax. Then, through a suitable speculum, carry the prepared tent through the cervix, and, if desirable, to the fundus, and let it remain twenty-four hours. No remedy in my hands has done more good in as short a time, in chronic inflammation, engorgement, enlargement or ulceration of the os and cervix uteri, and I have never known any unpleasant results from it.—*Dr. Wm. A. Gillespie, of Virginia.*

